# The second round of Critical Assessment of Automated Structure Determination of Proteins by NMR: CASD-NMR-2013

**DOI:** 10.1007/s10858-015-9953-4

**Published:** 2015-06-14

**Authors:** Antonio Rosato, Wim Vranken, Rasmus H. Fogh, Timothy J. Ragan, Roberto Tejero, Kari Pederson, Hsiau-Wei Lee, James H. Prestegard, Adelinda Yee, Bin Wu, Alexander Lemak, Scott Houliston, Cheryl H. Arrowsmith, Michael Kennedy, Thomas B. Acton, Rong Xiao, Gaohua Liu, Gaetano T. Montelione, Geerten W. Vuister

**Affiliations:** Department of Chemistry and Magnetic Resonance Center, University of Florence, 50019 Sesto Fiorentino, Italy; Structural Biology Brussels, Vrije Universiteit Brussel, Pleinlaan 2, 1050 Brussels, Belgium; (IB)2 Interuniversity Institute of Bioinformatics in Brussels, ULB-VUB, Triomflaan, 1050 Brussels, Belgium; Department of Biochemistry, School of Biological Sciences, University of Leicester, Henry Wellcome Building, Lancaster Road, Leicester, LE1 9HN UK; Departamento de Química Física, Universidad de Valencia, Avda. Dr. Moliner 50, 46100 Burjassot (Valencia), Spain; Complex Carbohydrate Research Center and Northeast Structural Genomics Consortium, University of Georgia, Athens, GA 30602 USA; Department of Medical Biophysics, Cancer Genomics and Proteomics, Ontario Cancer Institute, Northeast Structural Genomics Consortium, University of Toronto, Toronto, ON M5G 1L7 Canada; Department of Chemistry and Biochemistry, Northeast Structural Genomics Consortium, Miami University, Oxford, OH 45056 USA; Department of Molecular Biology and Biochemistry, Center for Advanced Biotechnology and Medicine, Northeast Structural Genomics Consortium, Rutgers, The State University of New Jersey, Piscataway, NJ 08854 USA; Department of Biochemistry and Molecular Biology, Robert Wood Johnson Medical School, Piscataway, NJ 08854 USA

**Keywords:** Protein, NMR, Structure determination, Automation, Quality, Validation, Blind testing, NOE, Chemical shift, CASD-NMR, Accuracy, Precision

## Abstract

The second round of the community-wide initiative Critical Assessment of automated Structure Determination of Proteins by NMR (CASD-NMR-2013) comprised ten blind target datasets, consisting of unprocessed spectral data, assigned chemical shift lists and unassigned NOESY peak and RDC lists, that were made available in both curated (i.e. manually refined) or un-curated (i.e. automatically generated) form. Ten structure calculation programs, using fully automated protocols only, generated a total of 164 three-dimensional structures (entries) for the ten targets, sometimes using both curated and un-curated lists to generate multiple entries for a single target. The accuracy of the entries could be established by comparing them to the corresponding manually solved structure of each target, which was not available at the time the data were provided. Across the entire data set, 71 % of all entries submitted achieved an accuracy relative to the reference NMR structure better than 1.5 Å. Methods based on NOESY peak lists achieved even better results with up to 100 % of the entries within the 1.5 Å threshold for some programs. However, some methods did not converge for some targets using un-curated NOESY peak lists. Over 90 % of the entries achieved an accuracy better than the more relaxed threshold of 2.5 Å that was used in the previous CASD-NMR-2010 round. Comparisons between entries generated with un-curated versus curated peaks show only marginal improvements for the latter in those cases where both calculations converged.

**Electronic supplementary material:**

The online version of this article (doi:10.1007/s10858-015-9953-4) contains supplementary material, which is available to authorized users.

## Introduction

Manual determination of a protein structure from nuclear magnetic resonance (NMR) spectroscopy data is a time-consuming process, taking weeks up to several months for unfavorable cases. Moreover, as several of the required steps in this process can be complicated, the researcher must possess a high level of skill in order to produce a high quality structure. First the chemical shifts (CSs) observed in multidimensional NMR experiments must be assigned specifically to the originating atom in the molecule, a process called resonance assignment. Next, the thousands of through-space dipolar coupling signals (nuclear Overhauser effects or NOEs) observed in multidimensional NOE spectroscopy (NOESY) experiments must be identified (peak picking). These unassigned NOE peaks are then assigned to interproton interactions in the molecule, and converted into interatomic distance restraints, a process commonly called NOESY assignment. Additional conformational restraints can be obtained also from residual dipolar couplings (RDCs), scalar couplings and/or CS data. Finally, specialized software uses these distance and/or conformational restraints to produce a set of protein conformations, denoted as a structure ensemble of conformers, that is consistent with the experimental data. In the last decade, considerable effort has been expended in automation of these processes (Herrmann et al. 2002a; Linge et al. 2003a; Huang et al. 2006; Donald and Martin 2009; Williamson and Craven 2009; Gossert et al. 2011; Guerry and Herrmann 2011; Güntert and Buchner 2015), with some of these only requiring CSs (Cavalli et al. 2007; Shen et al. 2008) and several strategies are now consistently producing results comparable to a skilled researcher.

In order to evaluate the ability of automated methods to produce protein structures that closely match structures manually determined by experts, the community-wide initiative Critical Assessment of Automated Structure Determination of Proteins by NMR (CASD-NMR) was launched in 2009 (Rosato et al. 2009). In the first round, seven research groups involved in the development of NMR structure determination tools were provided with ten “blind” data sets consisting of assigned CS lists and unassigned NOESY peak lists that had been manually curated (i.e. filtered to remove noise peaks) and asked to generate structures using fully automated protocols only, i.e. no manual intervention other than data preparation was allowed. The same data were also used to manually produce reference structures that were only revealed after the submission of the automatically generated entries. The results were encouraging, demonstrating that automated structure-determination methods are feasible and reliable, particularly when they use manually-curated NOESY peak lists (Rosato et al. 2012). Subsequently, we will refer to this effort as CASD-NMR-2010.

Here, we present the input data and summarize the results from a second round of CASD-NMR that has recently been completed with the aims of assessing the progress that has been made in the automation of structure-determination methods and investigating the need for the curation of the NOESY peak lists for accurate structure-determination. In this round, participants were again given ten “blind” data sets. The research groups were also given assigned CS lists and (for some targets) RDC data correlated with HSQC peak positions, and were asked to generate structures from expert curated unassigned NOESY peak lists (as in 2009), un-curated unassigned NOESY peak lists, or unprocessed spectral data, to produce structures for comparison with the reference structures manually determined from the same data. We will refer to this effort as CASD-NMR-2013 to distinguish from the earlier CASD-NMR-2010 effort.

For the discussion in this and the accompanying paper on the structure validation of the results (Ragan et al. 2015), we first introduce the following definitions: a target comprises the initial data set(s), the restraints generated from these data and the reference ensemble of manually generated conformers describing the three-dimensional structure of the protein of interest; entries denote individual solutions for a target automatically calculated by a specific program using a specific (sub)set of the available data, and comprise an ensemble of conformers and all restraints generated during the calculation of the ensemble.

## Description of the CASD-NMR-2013 targets

**Fig. 1:**
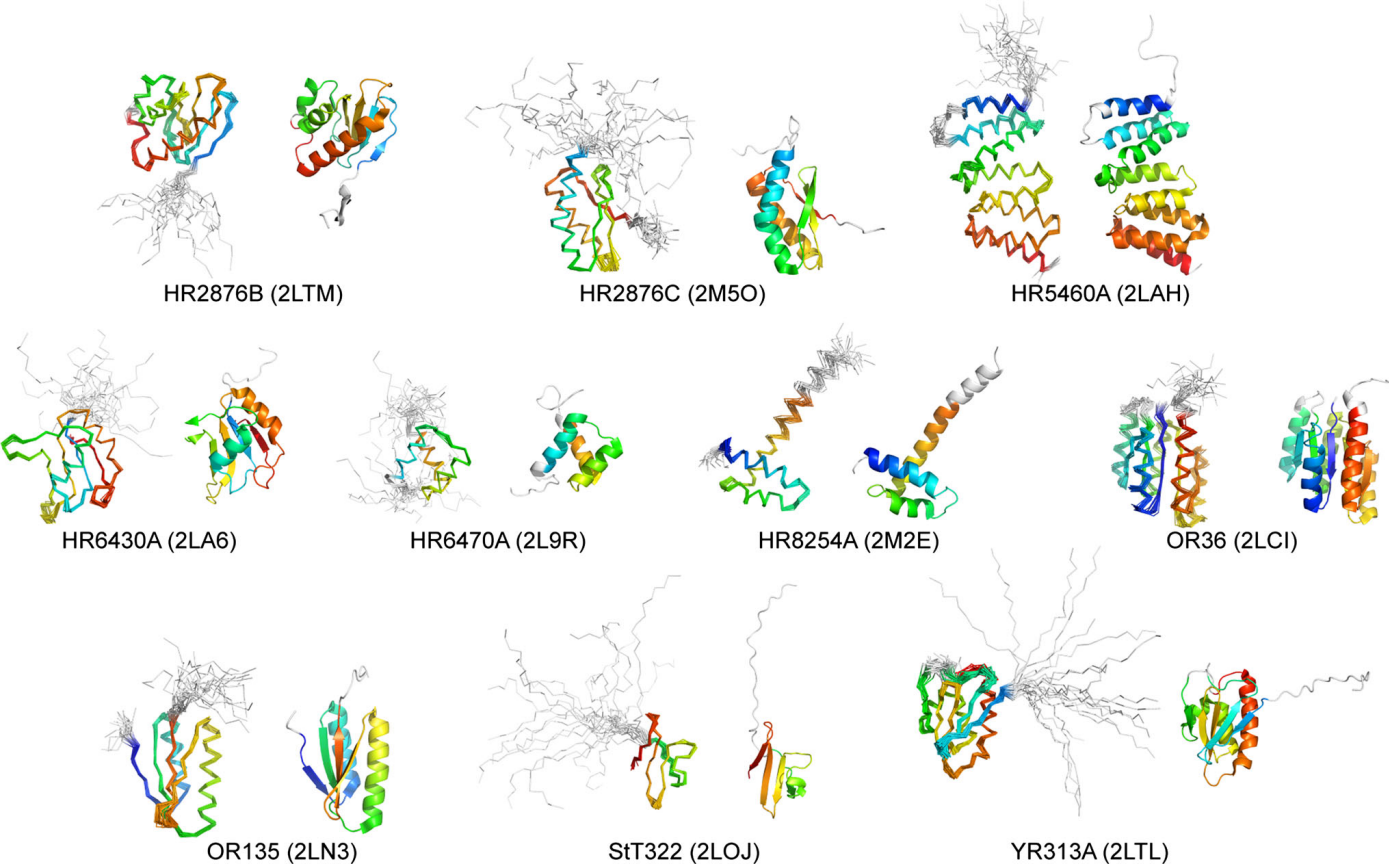
Side by side superimposed backbone ribbon traces and cartoon representations for the ten manually-determined CASD-NMR-2013 reference structures, labeled with PDB codes and coloured *blue* to *red* from N- to C-terminus. Ill-defined regions are shown in *light grey*

As the current effort was aimed at measuring progress since the CASD-NMR-2010 round and aimed to explore the effects of curated versus un-curated NOESY peak lists, the targets were again selected to comprise single domain, relatively small monomeric proteins, for which it is expected that high-resolution NMR spectroscopy can yield high-quality structures when using state-of-the-art technology. All targets were generated by the Northeast Structural Genomics (NESG) consortium of the National Institutes of Health Protein Structure Initiative (Huang et al. 2005a; Fig. 1 and Supplementary Table S1).

## Determination of the experimental structures of reference targets

### Protein expression and purification

Proteins were expressed and purified either at Rutgers University or the University of Toronto following the standard NESG protocols (Acton et al. 2005, 2011). These targets include six human protein domains: HR6470A, the homeobox domain of homeobox protein Nkx-3.1; HR6430A, the RRM domain of RNA-binding protein FUS; HR5460A, the N-terminal domain of mitotic checkpoint serine/threonine protein kinase BUB1, HR2876B and HR2876C, the N- and C-terminal domains of iron-sulfur cluster scaffold homolog NFU1; and HR8254A, the C terminal domain of DnaJ homolog subfamily C member 2. Besides targets from *Homo sapiens*, YR313A, the N-terminal NFU1 domain from yeast; StT322, a putative cytoplasmic protein ydiE; and two de novo designed ideal fold proteins (Koga et al. 2012), IF3 like fold design OR135 and Ploop2x3 fold design OR36, were used for the CASD-NMR-2013 study. *U*-^15^N, 5 %^13^C-enriched proteins and *U*-^15^N, *U*-^13^C-enriched proteins were expressed using MJ9 minimal media (Jansson et al. 1996). The *U*-^15^N, 5 %^13^C-labeled proteins were generated for stereo-specific assignments of isopropyl methyl groups of valines and leucines (Neri et al. 1989) and for residual dipolar coupling (RDC) measurements (Prestegard et al. 2004). The final purified protein samples prepared at Rutgers include a short N-terminal tag, with sequence MGH_6_SHM, for targets HR6470A, HR6430A, HR5460A, HR2876B, HR2876C and YR313A, or a short C-terminal LEHHHHHH tag for targets OR135 and OR36. The NMR samples of the two targets StT322 and HR8254 were prepared in Toronto and initially produced with larger purification tags, which were subsequently proteolytically removed to obtain the final NMR samples. The purified proteins were dissolved in 90 % ^1^H_2_O/10 % ^2^H_2_O NMR buffers optimized for the individual proteins.

### NMR data collection

All NMR spectra were recorded at 25 °C using cryogenic NMR probes at Rutgers University or University of Toronto. Triple resonance NMR data were collected on a Varian INOVA 600 MHz spectrometer or Bruker AVANCE 800 MHz spectrometers, while simultaneous 3D ^15^N/^13^C_aliphatic_/^13^C_aromatic_-edited NOESY (mixing time: 100 ms) and 3D ^13^C-edited aromatic NOESY (mixing time: 100 ms) spectra were acquired on a Bruker AVANCE 800 MHz spectrometer. 2D constant-time ^1^H–^13^C HSQC spectra, with 28 and 42 ms constant-time delays, were recorded for a *U*-^15^N, 5 %^13^C-enriched samples on the Varian INOVA 600 MHz spectrometer in order to obtain stereo-specific assignments for isopropyl groups of valines and leucines (Neri et al. 1989). Data recorded on targets StT322 and HR8254 used non-uniform sampling methods in the indirect dimensions (Orekhov et al. 2003; Gutmanas et al. 2002). All NMR data were processed using the program NMRPipe (Delaglio et al. 1995) and analysed using the program XEASY (Bartels et al. 1995). Spectra were referenced to external DSS. Sequence-specific resonance assignments were determined as described previously (Baran et al. 2004; Moseley et al. 2001). Targets StT322 and HR8254 employed the ABACUS semi-automated assignment strategy (Lemak et al. 2010). Chemical shift data were deposited in the Biological Magnetic Resonance Bank (Ulrich et al. 2008; cf. Table 1). Residual dipolar coupling measurements were made at University of Georgia on a 600 MHz Varian INOVA spectrometer. Samples were aligned with polyacrylamide gel or polyethylene-glycol-alkyl bicelles and RDCs were collected using either interleaved HSQC-TROSY or *J*-modulation sequences as described elsewhere (Eletsky et al. 2012; Lee et al. 2010).

**Table 1:** Targets and data of the CASD-NMR-2013 round

Target^1^	Protein length	PDB/BMRB codes	Secondary structure (% α/β/coil/ill-defined)	CS (#)	CS completion^2^ (%^1^H/^13^C/^15^N)	Un-curated NOESY peak lists (# peaks)			Curated NOESY peak lists (# peaks)			^1^H-^15^N RDCs (#)	r (#curated/#un-curated)^3^	Well-defined residue range(s)^4^	Closest homologue^5^	Homology (% identical/similar)^6^
						^13^C	^13^C-Arom.	^15^N	^13^C	^13^C-Arom.	^15^N					
HR2876B	107	2LTM/18489	23/22/41/13	1095	90/80/77	11,345	697	2060	5231	387	1436	121	0.50	13–105	2K1H	30/45
HR2876C	97	2M5O/19068	40/18/20/23	955	90/80/85	6870	217	2212	4580	161	1596	95	0.68	17–91	1VEH	93/97
HR5460A	160	2LAH/17524	65/1/20/14	1734	92/79/84	12,444	634	4172	8259	792	2964	83	0.70	14–25, 33–158	2WVI	39/60
HR6430A	99	2LA6/17508	19/28/39/13	975	89/80/81	4932	265	1628	4839	303	1501	126	0.97	14–99	2CPE	63/90
HR6470A	69	2L9R/17484	48/0/13/39	732	86/77/83	3103	169	990	3098	168	950	73	0.99	15–56	1NK2	69/79
HR8254A	73	2M2E/18909	56/0/19/25	859	96/88/89	15,073	421	3768	2549	163	853	None	0.19	554–608	2CQR	47/61
OR135	83	2LN3/18145	37/25/23/14	916	92/82/89	4669	143	2937	4680	150	1529	116	0.82	4–74	2L69	20/48
OR36	134	2LCI/17613	49/22/17/12	1558	94/84/91	10,846	314	2634	7125	209	2125	165	0.69	2–46, 53–125	1MEJ	27/33
StT322	63	2LOJ/18214	0/35/30/35	681	93/85/86	7454 3162^7^	28	1793	1596 270^7^	26	835	None	0.18	23–63	1ON4	34/54
YR313A	119	2LTL/18487	28/22/31/19	1259	91/81/87	10,171	148	1984	4897	90	1605	112	0.54	17–41, 45–115	2M6Q	19/39

^1^See Supplementary Table S1 for additional target information

^2^Assignments as a percentage of *all* proton signals, *all* carbon signals, and *backbone only* nitrogen signals, respectively. The totals include signals that are not assignable with standard experiments, such as proline backbone N and exchangeable protons

^3^Ratio of curated to un-curated peak count across all NOESY experiments used

^4^Well-defined ranges as determined by CyRange (Kirchner and Guentert 2011)

^5^Closest homologue in the PDB dated prior to the release date of the target. Where several entries had the same homology we have given priority to structure ensembles for systems and conditions as similar to the target as possible

^6^Percentage of identical/similar residues for the well-defined regions

^7^Recorded in D_2_O

### Manual NMR structure calculations

For NMR structure calculations, initial NOESY peak lists containing expected intra-residue, sequential, and α-helical medium-range NOE peaks were first generated from the resonance assignments and then manually edited by visual inspection of the NOESY spectra. Subsequent manual peak picking was then used to identify remaining peaks that were not easily identified from these simulated spectra, which arise primarily from long-range NOEs. Backbone dihedral angle constraints were derived from chemical shift data using the program TALOS+ (Shen et al. 2009) for residues located in well-defined secondary structure elements. The program CYANA (Herrmann et al. 2002b) was used to automatically assign NOEs and to calculate the NMR structure ensemble. Subsequently, RPF-DP analysis (Huang et al. 2012) was used to guide manual peak list editing, including iterative cycles of noise/artefact peak removal, peak picking and NOE assignments. The output of the RPF-DP program was used to iteratively refine the NOESY peaks list, followed by cycles of automated analysis and structure generation, together with RDC data, with CYANA. In the final cycle of structure generation calculations, the 20 conformers with the lowest target function value were refined with RDC data in the presence of explicit water solvent (Linge et al. 2003b) using the program CNS (Brunger et al. 1998). The resulting structure coordinates and restraints were deposited in the Protein Data Bank (Berman et al. 2003), and relevant metrics are summarized in Supplementary Table S2.

### Validation of the experimental NMR reference structures

NMR data statistics, structural statistics, and global structure quality factors including Verify3D (Lüthy et al. 1992), ProsaII (Sippl 1993), PROCHECK (Laskowski et al. 1993), and MolProbity (Chen et al. 2009) raw and statistical Z-scores were computed using the PSVS version 1.4 software package (Bhattacharya et al. 2006). The global goodness-of-fit of the final structure ensembles with the NOESY peak list data was determined using the RPF analysis program (Huang et al. 2005b, 2012). The NESG minimum standard scores for each global structure-quality score were used as an initial guide to assess the quality of each structure. In addition, a closer examination of the local structure quality was performed to identify potential problem areas. If either potential global or local structural problems were identified, then this information was reported back to the researcher performing the structure determination, and the researcher was asked to carefully reexamine the data and to resolve any problematic issues. The structural ensembles of the ten targets have excellent validation statistics, both with respect to structural criteria and model versus data. Superpositions of the NMR ensembles of the reference structures are shown in Fig. 1 and the structural statistics are presented in Supplementary Information (Table S2). A detailed analysis of the structural quality of the reference protein structures is also given in the accompanying paper (Ragan et al. 2015).

### Production of NOESY peak lists

Two sets of NOESY peak list data were provided for each protein target in CASD-NMR-2013: (1) initial automatically-picked un-curated (or raw) NOESY peak lists and (2) the final manually-curated NOESY peak lists used for generating the reference structures that are deposited in the PDB. These NOESY peak lists and the final chemical shifts were provided in both XEASY (CYANA) and Sparky formats. The following protocol was used to prepare these “un-curated raw” NOESY peaks lists. First, peak lists for 2D ^15^N-HSQC, aliphatic ^13^C-HSQC and aromatic ^13^C-HSQC spectra were simulated, without spectral folding, from the complete NMR resonance assignments. These resonance frequencies were then manually curated by comparison with 3D ^13^C, ^15^N-edited NOESY spectra using the interactive spectral visualization program XEASY. This process ensures good matching between the chemical shifts in these simulated HSQC spectra and the experimental NOESY peak list. These refined simulated HSQC peak lists were then used to guide automatic peak picking of the entire experimental NOESY spectrum. The “restricted peak picking” module of the program Sparky (Goddard and Keller, Sparky 3, University of California, San Francisco) was used to automatically peak pick the 3D ^13^C, ^15^N-edited NOESY spectra. The ^15^N, ^13^C and direct ^1^H tolerances were chosen based on data resolution; typically 0.3–0.4 ppm for ^15^N and ^13^C dimensions, and 0.02–0.03 ppm for the direct ^1^H dimension. For the indirect ^1^H dimension, the match tolerance was set to the spectral width, as all frequencies are possible. Additional details on this simple peak picking protocol are available at http://wiki.nesg.org.

## CASD-NMR-2013 data sets

The data sets for CASD-NMR-2013 comprised CS assignments in both version 2.1 and 3.1 of the NMRSTAR format (Markley et al. 2003) and unassigned NOESY peak lists in SPARKY and/or XEASY/CARA format. The data and their metadata were made available on the WeNMR (Wassenaar et al. 2012) CASD-NMR website (http://www.wenmr.eu/wenmr/casd-nmr). For all targets, raw NOESY spectral data were also made available. CS assignments, unrefined peak lists and raw data were released simultaneously to the participants, 6–8 weeks ahead of the release of the target structure from the PDB. Refined lists were released 4 weeks after the release of the initial unrefined data, so that the participants had a minimum of 2 weeks to use them for structure calculation. With this design, a period of 4 weeks was available to generate an entry with unrefined peak lists or raw data, and two to four additional weeks were available to generate an entry with refined lists, while the manually solved structure was on hold in the PDB. The entries were deposited directly by the participants into a password-protected database, again via the CASD-NMR website. An overview of all targets and the data made available to the CASD-NMR-2013 round is given in Table 1.

## Description of the CASD-NMR-2013 entries

Current structure generation protocols are based either on NOESY-based distance restraints, on CS data, or a combination of the two. In CASD-NMR-2013, the data were used by a total of twelve different automated protocols, subsequently referred to as ‘Programs’ for NMR structure generation. The CASD-NMR-2013 effort is a near complete representation of the array of computational methods currently available to the NMR spectroscopist for generating three-dimensional atomic-resolution structures from NMR data. Detailed descriptions of the different participating programs and of the protocols used to prepare the entries are available as published or in subsequent papers (Table 2).

**Table 2:** CASD-NMR2013 participants

Program	References
ASDP (CNS/Rosetta)	Huang et al. (2006, 2015)
Aria	Mareuil et al. (2015)
Autonoe	Zhang et al. (2014)
CHESHIRE (YAPP)	Cavalli and Vendruscolo (2015)
CS-HM-Rosetta	Thompson et al. (2012)
Cyana	Herrmann et al. (2002b), López-Méndez and Güntert (2006)
Ponderosa	Lee et al. (2011)
BE-Metadynamics	Granata et al. (2013)
i-TASSER	Jang et al. (2015)
Rosetta-web	van der Schot and Bonvin (2015)
UNIO	Guerry et al. (2015)

Programs submitting to CASD-NMR-2013 and references to the protocols used

**Fig. 2:**
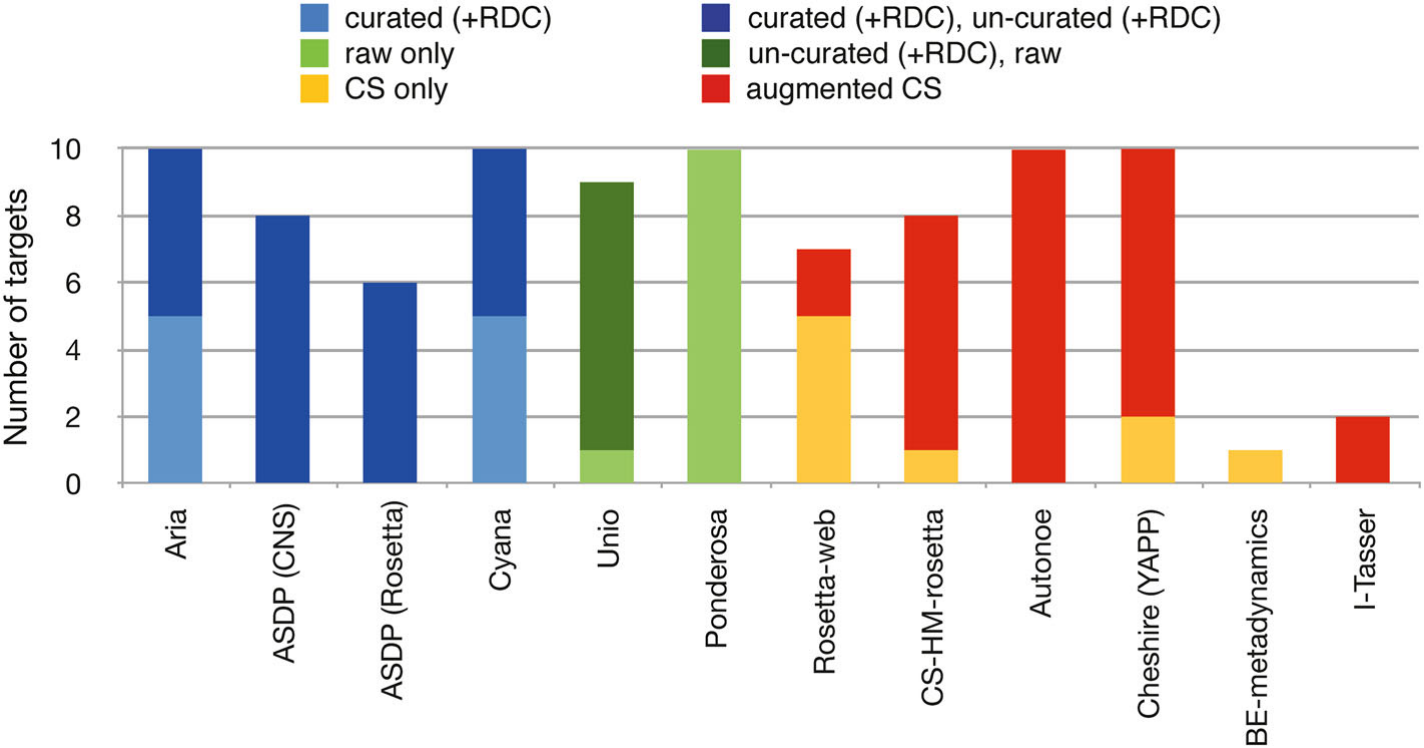
Targets submitted per program. A target is counted as submitted if there is at least one entry; programs often submitted multiple entries for a single target. Calculations that did not converge are ignored. Colour (see legend) encodes the targets calculated using different input data sets; e.g. using curated NOESY peak lists only (*light blue*) or two entries for one target with one using curated and one using curated peaklists (*dark blue*). Results for ASDP were provided using two different refinement methods (CNS and Rosetta), but at least one submission was provided for each of the 10 targets

A total of 164 entries were submitted covering all ten targets (Fig. 2). UNIO and Ponderosa were the only programs that submitted entries calculated directly from raw, unprocessed spectral data. However, not all programs submitted entries for all targets and not all the programs that used NOESY data submitted entries for both un-curated and curated peak lists. This could potentially distort the apparent success rates of the programs. Queries with the submitting groups revealed that 36 calculations that might otherwise have been expected had never been attempted (or, in a couple of cases, could not be carried out in time because of temporary file I/O problems). Eleven calculations were attempted but did not converge: Five ARIA and one Cheshire-YAPP entries for un-curated peaks were deposited but qualified as ‘incorrect’ by the submitters, and these served as controls, but were excluded from all analyses. A further three CYANA calculations started from unrefined peaks lists and two Rosetta web server calculations did not converge and were never deposited. Finally, nine entries from three different programs were missing without it being possible to determine the reason.

## Results and discussion

The results of CASD-NMR-2013 provide a comprehensive comparison of the performance of most currently available programs for automated protein structure generation from NMR data. We have evaluated both methods that primarily rely on NOESY peak lists as the input data and methods that use CS data as the primary input data. The latter are sometimes augmented by the use of the NOESY peak lists or RDCs, usually for the purpose of selection from the initially generated ensemble of structures. We also assessed the impact of using un-curated rather than curated NOESY peak lists. Furthermore, we have evaluated structures generated by methods that use CS data only (Cheshire and Rosetta) or raw spectral data (Ponderosa and UNIO) as the input data.

The wwPDB NMR Validation Task Force has recommended that the data for ill-defined regions should be part of the deposited dataset, but should not be included in the validation analysis (Montelione et al. 2013). It also recommended the program CyRange (Kirchner and Guentert 2011) as a robust tool for determining the well-defined and ill-defined regions. Following this recommendation, we established the well-defined regions for the ten targets (Table 1) and used these to superpose targets and entries. Assuming that the manually determined reference ensemble constitutes the correct representation of the three-dimensional structure of the target protein, the average pairwise backbone RMSD between the target ensemble and the entry ensemble were used as a measure of accuracy (RMSD bias). The use of CYANA to calculate the reference structures could in theory bias the results, but no such effect was evident from the data. A closer investigation would require recalculating structures with different programs using identical, manually curated input data. An entry is considered to be indistinguishable from the target when the average RMSD between the two ensembles is less than the sum of their ensemble convergence. We established a threshold of 1.5 Å to be a reasonable criterion, above which any ensembles can be regarded as describing structures with differences beyond experimental uncertainty (see Ragan et al. 2015). This threshold is somewhat more restrictive than that used to identify accurate structures in CASD-NMR-2010. We also used a slightly different method to compute the RMSD bias, i.e. as the average RMSD between the conformers in the two ensembles instead of the RMSD between the two average conformers, as was done for CASD-NMR-2010. Thus, a level of accuracy comparable to CASD-NMR-2010 is in between 2 and 2.5 Å.

**Fig. 3:**
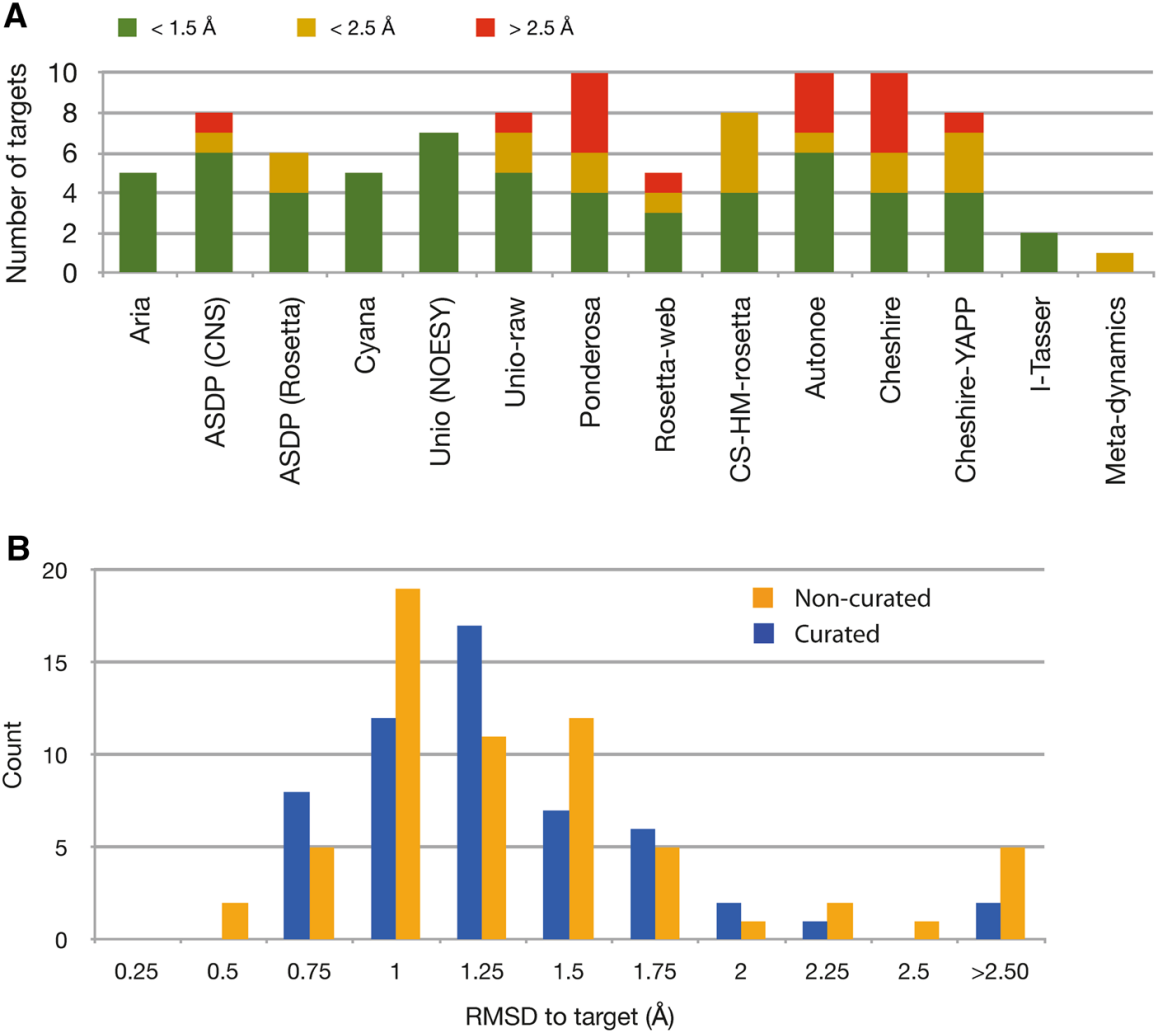
Accuracy of CASD-NMR-2013 entries based on un-curated information. **a** Number of CASD-NMR2013 entries based on un-curated NOESY, raw spectral data, or chemical shift (CS)-only data, colour-coded based on their accuracy. For each program, we include all targets for which there was at least one entry submitted. Each column is colour-coded based on the average accuracy of the submitted entry(ies) (compare to Table 2). *Green* high accuracy (RMSD bias to the reference <1.5 Å); *orange* intermediate accuracy (RMSD bias to the reference <2.5 Å); *red* low accuracy. **b** Histograms of the accuracy of entries using curated (*blue*, n = 55) or un-curated (*orange*, n = 63) peak lists. The RMSD values were calculated as the average of the pairwise root mean square deviation of the backbone atoms between the conformers in the target and entry ensembles using the well-defined regions as determined by CyRange (cf. Table 2)

Figure 3 shows the fraction of entries provided by each method using un-curated peak lists or raw data in relation to accuracy (cf. Table 3). Because of the similar outcomes, we grouped entries calculated with or without RDC restraints. The median accuracy over the entire dataset is 1.14 Å, with 71 % of the entries below the 1.5 Å threshold. Approaches using NOESY peak lists (curated or un-curated) achieved the best results, with a median accuracy of 1.07 Å and 80 % of the entries below the threshold (increasing up to 100 % for some programs). In contrast, calculations based on either raw spectral data or CS-only data performed less well, both approaches yielding a median accuracy of 1.5 Å with 50 % of ensembles below the threshold. These statistics are discussed in more detail in the accompanying paper (Ragan et al. 2015).

**Table 3:** Accuracy of CASD-NMR2013 entries based on un-curated NOESY, raw spectral data, or chemical shift (CS)-only data

Target	Reference	Based on un-curated NOESY lists					Raw spectra	
		ARIA	ASDP	CYANA	I-Tasser	UNIO	UNIO	Ponderosa
HR2876B	(0.62)	0.98 (0.64)	0.94 (0.68)			1.03 (0.45)	1.04 (0.58)	1.29 (0.11)
HR2876C	(0.53)	0.81 (0.42)	1.41 (0.65)	0.97 (0.30)	1.11 (n.a.)	1.12 (0.50)	1.32 (0.63)	0.95 (0.17)
HR5460A	(0.60)		1.52 (1.14)				2.26 (1.40)	9.33 (0.93)
HR6430A	(0.52)	0.82 (0.43)	0.95 (0.77)	0.90 (0.34)		0.91 (0.73)	1.13 (0.74)	1.03 (0.05)
HR6470A	(0.40)	0.56 (0.42)	1.00 (0.51)	0.59 (0.46)		0.66 (0.51)	1.09 (0.61)	0.66 (0.06)
HR8254A	(0.72)		1.73 (1.32)		1.31 (0.25)	1.45 (0.95)		3.01 (0.02)
OR135	(0.64)	0.90 (0.53)	0.96 (0.38)	0.98 (0.38)		0.98 (0.60)	1.01 (0.56)	1.91 (0.28)
OR36	(0.77)		1.34 (0.95)	1.14 (0.33)		1.43 (0.70)	1.57 (1.06)	2.78 (0.27)
StT322	(0.57)		2.56 (1.46)					3.69 (0.24)
YR313A	(0.97)		1.44 (0.93)				2.94 (1.82)	1.67 (0.11)
Median	n.a.	0.82	1.34	0.91	1.21	1.08	1.55	2.63
<1.5 Å	n.a.	5 (100 %)	7 (70 %)	5 (100 %)	2 (100 %)	7 (100 %)	5 (63 %)	4 (40 %)
<2.0 Å	n.a.	5 (100 %)	9 (90 %)	5 (100 %)	2 (100 %)	7 (100 %)	6 (75 %)	6 (60 %)
<2.5 Å	n.a.	5 (100 %)	9 (90 %)	5 (100 %)	2 (100 %)	7 (100 %)	7 (88 %)	6 (60 %)

Target	CS + un-curated NOESY		CS only			CS + RDCs
	Autonoe-Rosetta	Cheshire-YAPP	Cheshire	CS-Rosetta web server	BE-metadynamics	CS-HM-Rosetta
HR2876B	1.49 (1.17)	1.00 (0.48)	3.06 (3.2)			2.30 (n.a.)
HR2876C	1.16 (0.79)	2.07 (1.14)	1.84 (1.31)	0.98 (0.70)	1.68 (1.66)	1.88 (0.42)
HR5460A	3.46 (2.56)	5.79 (4.85)	3.52 (n.a.)	3.12 (2.9)		1.70 (1.33)
HR6430A	1.79 (1.82)	1.03 (0.75)	1.19 (n.a.)			1.21 (0.59)
HR6470A	0.81 (0.79)	0.64 (0.34)	0.81 (n.a.)	0.62 (0.46)		0.54 (0.32)
HR8254A	3.91 (0.97)		2.77 (n.a.)	1.52 (1.31)		1.38 (0.48)
OR135	1.01 (0.52)	1.57 (0.94)	1.29 (n.a.)	0.93 (0.59)		1.01 (0.74)
OR36	1.46 (1.24)	1.24 (0.91)	2.64 (n.a.)			
StT322	4.34 (0.44)		1.30 (0.82)			
YR313A	1.20 (0.75)	1.73 (1.53)	2.04 (n.a.)			2.17 (n.a.)
Median	1.48	1.41	1.94	0.98	1.68	1.54
<1.5 Å	6 (60 %)	4 (50 %)	4 (40 %)	3 (60 %)	0 (0 %)	4 (50 %)
<2.0 Å	7 (70 %)	6 (75 %)	5 (50 %)	4 (80 %)	1 (100 %)	6 (75 %)
<2.5 Å	7 (70 %)	7 (88 %)	6 (60 %)	4 (80 %)	1 (100 %)	8 (100 %)

Structure precision is also shown in parentheses. When multiple structures were submitted for a given target based on the same method, the average accuracy and precision are given. Structures marked as not converged or incorrect at the time of submission were excluded. At the bottom of the table, we report the median accuracy of each tool. In addition, the number of structures with an accuracy better than a fixed threshold are reported, together with the percentage of entries that were better than (i.e. correct within) the threshold. Tools were grouped according to the main input data used

CASD-NMR-2013 had a specific focus on the structure calculation performance achievable starting from automatically generated un-curated peak lists or raw spectral data, in addition to evaluating the progress of CS-only approaches. For the former set of calculations, in most cases the fraction of accurate entries (RMSD bias ≤1.5 Å) was 50 % or more of all submitted entries, up to 100 % for the NOESY-based methods (ARIA, CYANA UNIO; Table 3). Note however that some of these NOESY-based methods did not submit entries for all targets, precluding head-to-head comparisons of the methods for more than a few targets (Table 3). In absolute terms, NOESY-based methods provided accurate entries for up to seven of the ten targets using un-curated lists (ASDP; UNIO). This value increases to ten (ASDP) and a success rate of 100 % assuming a more relaxed accuracy threshold of ~2.5 Å, comparable to the previous CASD-NMR-2010 round (Rosato et al. 2012).

For approaches based on raw data, the performance is close to that observed for traditional methods, with UNIO performing better than Ponderosa. Chemical-shift based tools can be run using CS-only data, or in conjunction with additional un-curated NOESY or RDC peak lists to filter or bias the results obtained with CS data. Cheshire-YAPP and Autonoe-Rosetta, which integrate NOESY data, provided four and six correct structures, respectively, corresponding to 50 and 60 % of the submissions (Fig. 3a). The number of correct structures was lower for CS-only based calculations, ranging between three and four for the various tools. CS-HM-Rosetta, which exploits RDC’s and restraints derived from protein homology analysis, provided four accurate structures; all of its eight submissions were within 2.5 Å from the target structure. I-Tasser joined CASD-NMR at an advanced stage and thus could provide blind entries only for the two latest targets; both entries were accurate. BE-Metadynamics similarly participated only for the last target, yielding a structure with an accuracy of 1.68 Å. While it is difficult to compare these methods with other methods assessed because only a few targets were submitted, their results were included in our analysis to encourage the participation of a wide range of methods in the CASD-NMR experiment.

Figure 3b shows a comparison of accuracies of the entries obtained from either the use of un-curated NOESY peak lists or curated peak lists. It reveals that, in cases where results were submitted for both curated and un-curated peak lists, there is no advantage to using curated peak lists as the two distributions are overlapping with very similar median accuracies of 1.08 Å (79 % of entries below the threshold) and 1.05 Å (80 % of the entries below the threshold), respectively. It appears that the iterative procedures implemented in the programs are efficient at filtering the peaks for consistent information. It should be noted however, that five out of ten ARIA calculations and three out of eight CYANA calculations with un-curated peak lists failed to converge, where the corresponding calculations with curated peak lists converged to good quality structures. The convergence rate seems to depend on the quality of the un-curated peak lists. We estimated the quality of the un-curated peak list as the ratio r of the number of curated to un-curated peaks (Table 1). Three targets with r > 0.8 converged for both the CYANA and ARIA programs, and two targets with r < 0.2 failed to converge for both programs. The remaining five targets had 0.5 < r < 0.7; of these three failed to converge for ARIA, one failed to converge for CYANA, and two were not attempted by CYANA for unrelated reasons. All ten targets were submitted by ASDP, using either CNS or Rosetta refinement. We also analysed the relation between the number of un-curated peaks and structure accuracy. For the entries that do converge, the protein length and the number of assigned chemical shifts are only correlated with the final structure accuracy for the methods starting from raw data (Table S3), suggesting that these approaches are still sensitive to protein size. The number of unrefined peaks from the ^13^C and ^15^N NOESY, however, display a weak correlation with the final structure accuracy (Supplementary Figure S1 and Table S3). This is an additional indication that the combination of the quality and complexity of the NOESY spectra, which determines the number of un-curated NOESY peaks, may be relevant for the final structure accuracy.

Two of the targets were particularly challenging, StT322 and HR8254A. StT322 includes a large ill-defined region (Fig. 1), and HR8254A has a long third-helix extending outside the core, which makes RMSD metrics very sensitive to the precise determination of helical tilt angles (Fig. 1, see also Fig. 3 of Huang et al. 2015). These two data sets were the only ones to include non-uniform sampled NMR data, had the lowest ratio of curated to un-curated peaks (r < 0.2) and did not have RDC data (Table 1). Targets StT322 and HR8254A failed to converge in both CYANA and ARIA for un-curated peaks. Indeed, only one of the methods based on un-curated NOESY peaks lists, ASDP-Rosetta, reported results for both StT322 and HR8254A (Table 3); high RMSDs to these reference structures for the ASDP entries (2.56 and 1.73 Å) were the highest across all the methods based on un-curated NOESY peak lists (Table 3), significantly increasing the median RMSD score for ASDP results. Pondorosa, using raw spectra, also submitted results for StT322 and HR8254A with significantly higher RMSDs (3.69 and 3.01 Å, respectively) compared with most of the other Ponderosa results (Table 3). Entries submitted for the StT322 and HR8254A also had the worst accuracy scores among the targets submitted for Autonoe-Rosetta, while Cheshire did reasonably well with un-curated peaks provided for target StT322 (1.30 Å). As only a subset of the methods submitted results for these particularly challenging targets StT322 and HR8254A care should be taken in interpreting median RMSDs and percentages of structures in various accuracy ranges, summarized in Table 3, in comparing the performance of the several methods of CASD-NMR-2013.

In conclusion, the results from CASD-NMR-2013 demonstrate that for small, single domain proteins automated structure determination protocols are capable of reliably producing structures of comparable accuracy to those generated by a skilled researcher. Compared to CASD-NMR-2010, a significantly larger number of entries were successful and the overall accuracy was significantly better. As in the previous round, the performance of NOESY-based methods was superior to that of CS-only methods. Augmenting the latter with NOESY or other information resulted in an appreciable improvement. The present data show that the use of curated peak lists only yields marginal improvements over un-curated, automatically picked lists, provided that the calculations converge. High quality peak lists, with a ratio of curated to un-curated peaks above 0.5, greatly aids convergence, indicating that by using a conservative peak-picking approach the manual filtering step is no longer required for the successful application of the automated protocols, potentially broadening their utility. A more detailed comparison of the performance of individual programs can be found in the accompanying paper on validation of the structures (Ragan et al. 2015). The data collected for CASD-NMR-2013 will continue to be available as they provide a valuable resource for methods development in NMR (Bagaria et al. 2012; Lange 2014; Buchner and Güntert 2015). Subsequent rounds of CASD-NMR are expected to focus on automated structure determination from more challenging datasets e.g. for larger and/or multi-domain proteins, datasets containing various assignment errors or datasets lacking CS assignments. Another topic that merits further investigation is the incorporation of robust, fully automatic peak picking, as done in the present set of calculations by UNIO and PONDEROSA. We will also upgrade the data submission process for CASD-NMR and center it on the new NMR Exchange Format (NEF) (Gutmanas et al. 2015), which should facilitate the participation of a broader range of computational groups in future CASD-NMR experiments.

## Electronic supplementary material

Supplementary material 1 (PDF 449 kb)

## References

[CR1] Acton TB, Gunsalus KC, Xiao R, Ma L-C, Aramini J, Baran MC, Chiang Y-W, Climent T, Cooper B, Denissova NG, Douglas SM, Everett JK, Ho CK, Macapagal D, Rajan PK, Shastry R, Shih L-Y, Swapna GVT, Wilson M, Wu M, Gerstein M, Inouye M, Hunt JF, Montelione GT (2005) Robotic cloning and protein production platform of the northeast structural genomics consortium. In: sciencedirect.com. Elsevier, pp 210–243 10.1016/S0076-6879(05)94008-1 15808222

[CR55] Acton TB, Xiao R, Anderson S, Aramini JM, Buchwald W, Ciccosanti C, Conover K, Everett, JK, Hamilton K, Huang YJ, Janjua H, Kornhaber GJ, Lau J, Lee DY, Liu G, Maglaqui M, Ma LC, Mao L, Patel D, Rossi P, Sahdev S, Sharma S, Shastry R, Swapna GVT, Tang Y, Tong SN, Wang D, Wang H, Zhao L, Montelione GT (2011) Preparation of protein samples for NMR structure, function, and small molecule screening studies. Meth Enzymology 493:21–60. doi: 10.1016/B978-0-12-381274-2.00002-9 10.1016/B978-0-12-381274-2.00002-9 PMC4110644 21371586

[CR2] Bagaria A, Jaravine V, Huang YJ, Montelione GT, Güntert P (2012). Protein structure validation by generalized linear model root-mean-square deviation prediction. Protein Sci.

[CR3] Baran MC, Huang YJ, Moseley HNB, Montelione GT (2004). Automated analysis of protein NMR assignments and structures. Chem Rev.

[CR4] Bartels C, Xia T-H, Billeter M, Güntert P, Wüthrich K (1995). The program XEASY for computer-supported NMR spectral analysis of biological macromolecules. J Biomol NMR.

[CR5] Berman H, Henrick K, Nakamura H (2003). Announcing the worldwide Protein Data Bank. Nat Struct Biol.

[CR6] Bhattacharya A, Tejero R, Montelione GT (2006). Evaluating protein structures determined by structural genomics consortia. Proteins.

[CR7] Brunger AT, Adams PD, Clore GM, DeLano WL, Gros P, Grosse-Kunstleve RW, Jiang JS, Kuszewski J, Nilges M, Pannu NS, Read RJ, Rice LM, Simonson T, Warren GL (1998). Crystallography & NMR system: a new software suite for macromolecular structure determination. Acta Crystallogr D Biol Crystallogr.

[CR8] Buchner L, Güntert P (2015). Increased reliability of nuclear magnetic resonance protein structures by consensus structure bundles. Struct/Fold Des.

[CR9] Cavalli A, Vendruscolo M (2015). Analysis of the performance of the CHESHIRE and YAPP methods at CASD-NMR round 3. J Biomol NMR.

[CR10] Cavalli A, Salvatella X, Dobson CM, Vendruscolo M (2007). Protein structure determination from NMR chemical shifts. Proc Natl Acad Sci USA.

[CR11] Chen VB, Arendall WB, Headd JJ, Keedy DA, Immormino RM, Kapral GJ, Murray LW, Richardson JS, Richardson DC (2009). MolProbity: all-atom structure validation for macromolecular crystallography. Acta Crystallogr D Biol Crystallogr.

[CR12] Delaglio F, Grzesiek S, Vuister GW, Zhu G, Pfeifer J, Bax A (1995). NMRPipe: a multidimensional spectral processing system based on UNIX pipes. J Biomol NMR.

[CR13] Donald BR, Martin J (2009). Automated NMR assignment and protein structure determination using sparse dipolar coupling constraints. Prog Nucl Mag Res Sp.

[CR14] Eletsky A, Jeong M-Y, Kim H, Lee H-W, Xiao R, Pagliarini DJ, Prestegard JH, Winge DR, Montelione GT, Szyperski T (2012). Solution NMR structure of yeast succinate dehydrogenase flavinylation factor Sdh5 reveals a putative Sdh1 binding site. Biochemistry.

[CR15] Gossert AD, Hiller S, Fernández C (2011). Automated NMR resonance assignment of large proteins for protein–ligand interaction studies. J Am Chem Soc.

[CR57] Granata D, Camilloni C, Vendruscolo M, Laio A (2013) Characterization of the free-energy landscapes of proteins by NMR-guided metadynamics. Proc Natl Acad Sci USA 110:6817–6822. doi:10.1073/pnas.1218350110 10.1073/pnas.1218350110 PMC3637744 23572592

[CR16] Guerry P, Herrmann T (2011). Advances in automated NMR protein structure determination..

[CR17] Guerry P, Duong VD, Herrmann T (2015). CASD-NMR 2: robust and accurate unsupervised analysis of raw NOESY spectra and protein structure determination with UNIO. J Biomol NMR.

[CR18] Güntert P, Buchner L (2015). Combined automated NOE assignment and structure calculation with CYANA. J Biomol NMR.

[CR19] Gutmanas A, Jarvoll P, Orekhov VY, Billeter M (2002). Three-way decomposition of a complete 3D 15N-NOESY-HSQC. J Biomol NMR.

[CR20] Gutmanas A, Adams PD, Bardiaux B, Berman HM, Case D, Fogh RH, Guentert P, Hendrickx PMS, Herrmann T, Kleywegt G, Kobayashi N, Lange OF, Markley JK, Montelione GT, Nilges M, Ragan TJ, Schwieters CD, Tejero R, Ulrich E, Velankar S, Vranken WF, Wedell J, Westbrook J, Wishart DS, Vuister GW (2015) NMR exchange format: a unified and open standard for representation of NMR restraint data. Nat Struct Mol Biol 22:433–434. doi:10.1038/nsmb.3041 10.1038/nsmb.3041 PMC4546829 26036565

[CR21] Herrmann T, Güntert P, Wüthrich K (2002). Protein NMR structure determination with automated NOE-identification in the NOESY spectra using the new software ATNOS. J Biomol NMR.

[CR22] Herrmann T, Güntert P, Wüthrich K (2002). Protein NMR structure determination with automated NOE assignment using the new software CANDID and the torsion angle dynamics algorithm DYANA. J Mol Biol.

[CR23] Huang YJ, Moseley HNB, Baran MC, Arrowsmith C, Powers R, Tejero R, Szyperski T, Montelione GT (2005a) An integrated platform for automated analysis of protein NMR structures. In: sciencedirect.com. Elsevier, pp 111–141 10.1016/S0076-6879(05)94005-6 15808219

[CR24] Huang YJ, Powers R, Montelione GT (2005). Protein NMR recall, precision, and F-measure scores (RPF scores): structure quality assessment measures based on information retrieval statistics. J Am Chem Soc.

[CR25] Huang YJ, Tejero R, Powers R, Montelione GT (2006). A topology-constrained distance network algorithm for protein structure determination from NOESY data. Proteins.

[CR26] Huang YJ, Rosato A, Singh G, Montelione GT (2012). RPF: a quality assessment tool for protein NMR structures. Nucleic Acids Res.

[CR56] Huang YJ, Mao B, Xu F, Montelione GT (2015) Guiding automated NMR structure determination using a global optimization metric, the NMR DP score. J Biomol NMR. doi:10.1007/s10858-015-9955-2 10.1007/s10858-015-9955-2 PMC4943320 26081575

[CR27] Jang R, Wang Y, Xue Z, Zang Y (2015). NMR data-driven structure determination using NMR-I-TASSER in the CASD-NMR experiment. J Biomol NMR.

[CR28] Jansson M, Li Y-C, Jendeberg L, Anderson S, Montelione G, Nilsson BR (1996). High-level production of uniformly 15N-and 13C-enriched fusion proteins in *Escherichia coli*. J Biomol NMR.

[CR29] Kirchner DK, Guentert P (2011). Objective identification of residue ranges for the superposition of protein structures. BMC Bioinform.

[CR30] Koga N, Tatsumi-Koga R, Liu G, Xiao R, Acton TB, Montelione GT, Baker D (2012). Principles for designing ideal protein structures. Nature.

[CR31] Lange OF (2014). Automatic NOESY assignment in CS-RASREC-Rosetta. J Biomol NMR.

[CR32] Laskowski RA, MacArthur MW, Moss DS, Thornton JM (1993). PROCHECK: a program to check the stereochemical quality of protein structures. J Appl Cryst.

[CR33] Lee H-W, Wylie G, Bansal S, Wang X, Barb AW, Macnaughtan MA, Ertekin A, Montelione GT, Prestegard JH (2010). Three-dimensional structure of the weakly associated protein homodimer SeR13 using RDCs and paramagnetic surface mapping. Protein Sci.

[CR58] Lee W, Kim JH, Westler WM, Markley JL (2011) PONDEROSA, an automated 3D-NOESY peak picking program, enables automated protein structure determination. Bioinformatics 27:1727–1728 10.1093/bioinformatics/btr200 PMC3106192 21511715

[CR34] Lemak A, Gutmanas A, Chitayat S, Karra M, Farès C, Sunnerhagen M, Arrowsmith CH (2010). A novel strategy for NMR resonance assignment and protein structure determination. J Biomol NMR.

[CR35] Linge JP, Habeck M, Rieping W, Nilges M (2003a) ARIA: automated NOE assignment and NMR structure calculation. Bioinformatics 19:315–316 10.1093/bioinformatics/19.2.315 12538267

[CR36] Linge JP, Williams MA, Spronk CAEM, Bonvin AMJJ, Nilges M (2003). Refinement of protein structures in explicit solvent. Proteins.

[CR101] López-Méndez B, Güntert P (2006) Automated protein structure determination from NMR spectra. J Am Chem Soc 128:13112–13122 10.1021/ja061136l 17017791

[CR37] Lüthy R, Bowie JU, Eisenberg D (1992). Assessment of protein models with three-dimensional profiles. Nature.

[CR38] Mareuil F, Malliavin TE, Nilges M, Bardiaux B (2015). Improved reliability, accuracy and quality in automated NMR structure calculation with ARIA. J Biomol NMR.

[CR39] Markley JL, Ulrich EL, Westler WM, Volkman BF (2003). Macromolecular structure determination by NMR spectroscopy. Methods Biochem Anal.

[CR40] Montelione GT, Nilges M, Bax A, Güntert P, Herrmann T, Richardson JS, Schwieters CD, Vranken WF, Vuister GW, Wishart DS, Berman HM, Kleywegt GJ, Markley JL (2013). Recommendations of the wwPDB NMR validation task force. Structure.

[CR41] Moseley HNB, Monleon D, Montelione GT (2001) Automatic determination of protein backbone resonance assignments from triple resonance nuclear magnetic resonance data. In: sciencedirect.com. Elsevier, pp 91–108 10.1016/s0076-6879(01)39311-4 11462827

[CR42] Neri D, Szyperski T, Otting G, Senn H, Wüthrich K (1989). Stereospecific nuclear magnetic resonance assignments of the methyl groups of valine and leucine in the DNA-binding domain of the 434 repressor by biosynthetically directed fractional 13C labeling. Biochemistry.

[CR43] Orekhov VY, Ibraghimov I, Billeter M (2003). Optimizing resolution in multidimensional NMR by three-way decomposition. J Biomol NMR.

[CR44] Prestegard JH, Bougault CM, Kishore AI (2004). Residual dipolar couplings in structure determination of biomolecules. Chem Rev.

[CR45] Ragan TJ, Fogh RH, Tejero R, Vranken W, Montelione GT, Rosato A, Vuister GW (2015) Analysis of the structural quality of the CASD-NMR 2013 entries. J Biomol NMR. doi:10.1007/s10858-015-9949-0 10.1007/s10858-015-9949-0 PMC4569653 26032236

[CR46] Rosato A, Bagaria A, Baker D, Bardiaux B, Cavalli A, Doreleijers JF, Giachetti A, Guerry P, Güntert P, Herrmann T, Huang YJ, Jonker HRA, Mao B, Malliavin TE, Montelione GT, Nilges M, Raman S, van der Schot G, Vranken WF, Vuister GW, Bonvin AMJJ (2009). CASD-NMR: critical assessment of automated structure determination by NMR. Nat Methods.

[CR47] Rosato A, Aramini JM, Arrowsmith C, Bagaria A, Baker D, Cavalli A, Doreleijers JF, Eletsky A, Giachetti A, Guerry P, Gutmanas A, Güntert P, He Y, Herrmann T, Huang YJ, Jaravine V, Jonker HRA, Kennedy MA, Lange OF, Liu G, Malliavin TE, Mani R, Mao B, Montelione GT, Nilges M, Rossi P, van der Schot G, Schwalbe H, Szyperski TA, Vendruscolo M, Vernon R, Vranken WF, de Vries S, Vuister GW, Wu B, Yang Y, Bonvin AMJJ (2012). Blind testing of routine, fully automated determination of protein structures from NMR data. Structure.

[CR48] Shen Y, Lange OF, Delaglio F, Rossi P, Aramini JM, Liu G, Eletsky A, Wu Y, Singarapu KK, Lemak A, Ignatchenko A, Arrowsmith CH, Szyperski T, Montelione GT, Baker D, Bax A (2008). Consistent blind protein structure generation from NMR chemical shift data. Proc Natl Acad Sci USA.

[CR49] Shen Y, Delaglio F, Cornilescu G, Bax A (2009). TALOS+: a hybrid method for predicting protein backbone torsion angles from NMR chemical shifts. J Biomol NMR.

[CR50] Sippl MJ (1993). Recognition of errors in three-dimensional structures of proteins. Proteins.

[CR59] Thompson J, Sgourakis NG, Liu G, Rossi P, Tang Y, Mills J, Szyperski T, Montelione G, Baker D (2012) Accurate protein structure modeling using sparse NMR data and homologous structure information. Proc Natl Acad Sci USA 109:9875–9880 10.1073/pnas.1202485109 PMC3382498 22665781

[CR51] Ulrich EL, Akutsu H, Doreleijers JF, Harano Y, Ioannidis YE, Lin J, Livny M, Mading S, Maziuk D, Miller Z, Nakatani E, Schulte CF, Tolmie DE, Wenger RK, Yao H, Markley JL (2008). BioMagResBank. Nuclic Acids Res.

[CR52] van der Schot G, Bonvin AMJJ (2015). Performance of the WeNMR CS-Rosetta3 web server in CASD-NMR. J Biomol NMR.

[CR53] Wassenaar TA, Dijk M, Loureiro-Ferreira N, Schot G, Vries SJ, Schmitz C, Zwan J, Boelens R, Giachetti A, Ferella L, Rosato A, Bertini I, Herrmann T, Jonker HRA, Bagaria A, Jaravine V, Güntert P, Schwalbe H, Vranken WF, Doreleijers JF, Vriend G, Vuister GW, Franke D, Kikhney A, Svergun DI, Fogh RH, Ionides J, Laue ED, Spronk CAEM, Jurkša S, Verlato M, Badoer S, Dal Pra S, Mazzucato M, Frizziero E, Bonvin AMJJ (2012). WeNMR: structural biology on the grid. J Grid Comput.

[CR54] Williamson MP, Craven CJ (2009). Automated protein structure calculation from NMR data. J Biomol NMR.

[CR60] Zhang Z, Porter J, Tripsianes K, Lange OF (2014) Robust and highly accurate automatic NOESY assignment and structure determination with Rosetta. J Biomol NMR 59:135–145. doi:10.1007/s10858-014-9832-4 10.1007/s10858-014-9832-4 24845473

