# Supplementary material for: The second round of Critical Assessment of Automated Structure Determination of Proteins by NMR: CASD-NMR-2013
Source: J Biomol NMR. 2015 Jun 14;62(4):413–24. doi: 10.1007/s10858-015-9953-4 (PMC4569658; doi:10.1007/s10858-015-9953-4)
Supplement: Supplementary file 1 — Supplementary material 1 (PDF 449 kb) [file 10858_2015_9953_MOESM1_ESM.pdf]

The Second Round of Critical Assessment of Automated Structure Determination of Proteins by NMR: CASD-NMR-2013

Supplementary Information

Antonio Rosato[1&], Wim Vranken[2&], Rasmus H. Fogh[3&], Timothy J. Ragan[3], Roberto Tejero[4] James Prestegard[5], Adlinda Yee[6], Cheryl Arrowsmith[6], Michael Kennedy[7], Thomas B. Acton[8], Rong Xiao[8], Gaohua Liu[8], Gaetano T. Montelione[8*] and Geerten W. Vuister[3*]

[1] Magnetic Resonance Center, Department of Chemistry, University of Florence, 50019, Sesto Fiorentino, Italy.

[2] Structural Biology Brussels, Pleinlaan 2, Vrije Universiteit Brussel and (IB)[2] Interuniversity Institute of Bioinformatics in Brussels, ULB-VUB, Triomflaan, 1050 Brussels, Belgium.

[3] Department of Biochemistry, School of Biological Sciences, University of Leicester, Henry Wellcome building, Lancaster Road, Leicester, LE1 9HN, United Kingdom.

[4] Departamento de Química Física, Universidad de Valencia, Avda. Dr. Moliner 50. 46100 Burjassot (Valencia), Spain.

[5] Complex Carbohydrate Research Center and Northeast Structural Genomics Consortium, University of Georgia, Athens, Georgia 30602, USA.

[6]Cancer Genomics & Proteomics, Department of Medical Biophysics, Ontario Cancer Institute, and Northeast Structural Genomics Consortium, University of Toronto, Toronto, Ontario, M5G 1L7, Canada.

[7]Department of Chemistry and Biochemistry, Northeast Structural Genomics Consortium, Miami University, Oxford, Ohio 45056, USA.

[8] Center for Advanced Biotechnology and Medicine, Department of Molecular Biology and Biochemistry, and Northeast Structural Genomics Consortium, Rutgers, The State University of New Jersey, and Robert Wood Johnson Medical School, Piscataway, NJ 08854, USA.

[&] Equal contributions
[*] To whom correspondence can be addressed (gv29@le.ac.uk or guy@cabm.rutgers.edu)

Supplementary Table S1. Experimental protein NMR data sets.

NESG ID	Protein Uniprot ID	PDB DOI	PDB_ID	BMRB_ID	PDB Author List
HR6470A	NKX31_HUMAN	10.2210/pdb2l9r/pdb	2L9R	17484	Liu, G., Xiao, R., Lee, H.-W., Hamilton, K., Ciccosanti, C., Wang, H.B., Acton, T.B., Everett, J.K., Huang, Y.J., Montelione, G.T.
HR6430A	FUS_HUMAN	10.2210/pdb2la6/pdb	2LA6	17508	Liu, G., Xiao, R., Janjua, H., Lee, H., Ciccosanti, C.T., Acton, T.B., Everett, J.K., Huang, Y.J., Montelione, G.T.
OR36	de novo design	10.2210/pdb2lci/pdb	2LCI	17613	Liu, G., Koga, N., Koga, R., Xiao, R., Lee, H.T., Janjua, H., Ciccosanti, C., Acton, T.B., Everett, J., Baker, D., Montelione, G.T.
OR135	de novo design	10.2210/pdb2ln3/pdb	2LN3	18145	Liu, G., Koga, R., Koga, N., Xiao, R., Lee, H., Janjua, H., Kohan, E., Acton, T.B., Everett, J.K., Baker, D., Montelione, G.T.,
HR5460A	BUB1_HUMAN	10.2210/pdb2lah/pdb	2LAH	17524	Liu, G., Shastry, R., Ciccosanti, C., Hamilton, K., Acton, T.B., Xiao, R., Everett, J.K., Montelione, G.T.
StT322	Q7CQN6_SALTY	10.2210/pdb2loj/pdb	2LOJ	18214	Wu, B., Yee, A., Houliston, S., Garcia, M., Savchenko, A., Arrowsmith, C.H.
HR2876B	NFU1_HUMAN	10.2210/pdb2ltm/pdb	2LTM	18489	Liu, G., Xiao, R., Janjua, H., Hamilton, K., Shastry, R., Kohan, E., Acton, T.B., Everett, J.K., Lee, H., Huang, Y.J., Montelione, G.T.
YR313A	NFU1_YEAST	10.2210/pdb2ltl/pdb	2LTL	4986	Liu, G., Xiao, R., Hamilton, K., Janjua, H., Shastry, R., Kohan, E., Acton, T.B., Everett, J.K., Lee, H., Huang, Y.J., Montelione, G.T.
HR8254A	DNJC2_HUMAN	10.2210/pdb2m2e/pdb	2M2E	18909	Lemak, A., Yee, A., Houliston, S., Garcia, M., Ong, M., Arrowsmith, C.
HR2876C	NFU1_HUMAN	10.2210/pdb2m5o/pdb	2M5O	19068	Liu, G., Xiao, R., Janjua, H., Hamilton, K., Shastry, R., Kohan, E., Acton, T.B., Everett, J.K., Pederson, K., Huang, Y.J., Montelione, G.T.

Supplementary Table S2. Protein Structure Validation Server (PSVS) NMR structure quality statistics for ten CASD-NMR-2015 reference structures*

NESGID	**HR6470A**	**HR6430A**	**OR36**	**OR135**	**HR5460A**
PDBID	**2l9r**	**2la6**	**2lci**	**2ln3**	**2lah**
**NMR distance and dihedral constraints**					
Distance constraints					
Total NOE	1512	2844	3469	2711	4690
Intra-residue	402	534	881	576	971
Inter-residue					
Sequential (|i-j| = 1)	357	650	732	613	1030
Medium-range (|i-j| ≤ 4)	438	465	776	577	1340
Long-range (|i-j| ≥ 5)	315	1195	1080	945	1349
Intermolecular					
Hydrogen bonds	30	30	58	70	82
Total dihedral angle restraints	70	80	196	108	220
phi	35	40	98	54	110
psi	35	40	98	54	110
Total RDCs					
alignment media 1[§]	37	61	78	53	
alignment media 2[§]	37	65	87	51	
Number of restricting restraints per residue					
total	26.9	33.2	28.9	38.5	33.5
long range	5.2	13.6	8.4	12.9	9.1
**Structure statistics**					
Violations					
RMS of distance violation/constraint[¶] (Å)	0.01	0.01	0.01	0.01	0.01
RMS of dihedral angle violation/constraint (°)	0.10	0.84	0.61	0.63	0.26
Max distance constraint violation (Å)	0.20	0.33	0.31	0.51	0.40
Max dihedral angle violation (°)	1.70	9.5	7.1	5.7	5.0
Average mediod r.m.s.d.** (Å)					
Heavy	0.40±0.08	0.47±0.05	0.74±0.12	0.50±0.09	0.59±0.08
Backbone	1.07±0.05	1.00±0.08	1.37±0.11	1.15±0.10	1.08±0.07
RPF Scores					
Recall	0.993	0.986	0.986	0.972	0.961

Precision	0.962	0.977	0.967	0.972	0.96
F-measure	0.977	0.981	0.976	0.972	0.961
DP-scores	0.904	0.938	0.885	0.904	0.872
Structure quality factors (raw/Z-score)					
Procheck G-factor (phi / psi only)**	0.35/1.69	-0.16/-0.31	0.15/0.90	0.00/0.31	0.30/1.49
Procheck G-factor (all dihedral angles)**	0.30/1.77	-0.05/-0.30	0.07/0.41	0.07/0.41	0.14/0.83
Verify3D	0.22/-3.85	0.40/-0.96	0.54/1.28	0.40/-0.96	0.44/-0.32
ProsaII (-ve)	0.52/-0.54	0.84/0.79	1.40/3.10	0.86/0.87	0.89/0.99
MolProbity clashscore	13.72/-0.83	12.06/-0.69	18.92/-1.72	13.59/-0.81	16.81/-1.36
Ramachandran plot summary from Richardson's lab					
Most favored regions (%)	99.6	98.6	99.1	99.5	98.5
Allowed regions (%)	0.4	1.4	0.9	0.5	.1.5
Disallowed regions (%)	0	0	0	0.0	0

Supplementary Table S2 (con't)

NESGID	StT322	HR2876B	YR313A	HR8254A	HR2876C
PDBID	**2loj**	**2ltm**	**2ltl**	**2m2e**	**2m5o**
**NMR distance and dihedral constraints**					
Distance constraints					
Total NOE	933	2799	2319	1402	2857
Intra-residue	204	507	588	292	556
Inter-residue					
Sequential (|i-j| = 1)	257	676	604	366	701
Medium-range (|i-j| ≤ 4)	127	493	391	444	711
Long-range (|i-j| ≥ 5)	345	1123	736	300	889
Intermolecular					
Hydrogen bonds	36	50	40		46
Total dihedral angle restraints	70	112	122	125	120
phi	35	56	61		60
psi	35	56	61		60
Total RDCs					
alignment media 1§		60	55		46
alignment media 2§		61	59		49
Number of restricting restraints per residue					
total	17.0	30.5	23.2	21.5	34.4
long range	6.1	11.8	7.0	4.2	10.3
**Structure statistics**					
Violations					
RMS of distance violation/constraint¶ (Å)	0.02	0.01	0.01	0.01	0.01
RMS of dihedral angle violation/constraint (°)	0.27	0.67	0.63	0.30	0.63
Max distance constraint violation (Å)	0.41	0.43	0.32	0.28	0.34
Max dihedral angle violation (°)	2.50	7.30	6.3	2.10	6.7
Average mediod r.m.s.d.** (Å)					
Heavy	0.49±0.07	0.57±0.07	0.75±0.08	1.20±0.23	0.48±0.05
Backbone	1.17±0.10	1.15±0.08	1.33±0.09	1.80±0.16	0.85±0.07
RPF Scores					
Recall	0.958	0.988	0.987	0.962	0.959
Precision	0.921	0.969	0.953	0.951	0.975
F-measure	0.939	0.979	0.97	0.956	0.967

DP-scores	0.805	0.929	0.869	0.793	0.893
Structure quality factors (raw/Z-score)					
Procheck G-factor (phi / psi only)**	-0.82/-2.91	-0.09/-0.04	-0.22/-0.55	0.33/1.61	-0.05/0.12
Procheck G-factor (all dihedral angles)**	-0.68/-4.02	-0.05/-0.30	-0.10/-0.59	0.18/1.06	0.04/0.24
Verify3D	0.12/-5.46	0.40/-0.96	0.29/-2.73	0.29/-2.73	0.35/-1.77
ProsaII (-ve)	0.21/-1.82	0.80/0.62	0.52/-0.54	-/-	0.57/-0.33
MolProbity clashscore	9.23/-0.06	8.76/0.02	12.26/-0.58	11.72/-0.49	12.59/-0.63
Ramachandran plot summary from Richardson's lab					
Most favored regions (%)	92.3	97.4	98.9	98.6	98.7
Allowed regions (%)	7.7	2.6	1.1	1.4	1.3
Disallowed regions (%)	0	0	0	0.0	0

* Analyzed for the 20 lowest energy refined NMR structures of each target, by using PDBSTAT and PSVS 1.4[23 24].

§ PEG and phage were used as alignment media 1 and 2.

¶ Calculated by using sum over r^{-6}.

** Calculated among 20 refined structures for well-defined residues that have sum of phi and psi order parameters[3] S(phi)+S(psi)>1.8[1]. The well-defined residues of HR6470A: 11-58; HR6340A: 13-87, 90-98; HR5460a: 12-27, 33-158; OR36: 3-47, 53-128; OR135: 5-64, 67-73; StT322: 24-62; YR313A: 17-26,29-34,45-111; HR2876B: 12-58,61-67,70-106; HR8254A: 551-620; HR2876C: 17-48,51-58,64-93. RMSD value were calculated by MOLMOL, the medoid structure of each targets are: HR6470A, the 3rd conformer; HR6430A, the 15th conformer, HR5460A, the 8th conformer; OR36, the 19th conformer; OR135, the 5th conformer; StT322, the 19th conformer; YR313A, the 1st conformer; HR2876B, the 9th conformer; HR8254A, the 4th conformer; HR2876C, the 16th conformer.

⌘ With respect to mean and standard deviation for a set of 252 X-ray structures with sequence lengths < 500, resolution ≤ 1.80 Å, R-factor ≤ 0.25 and R-free ≤ 0.28; a positive value indicates a 'better' score.

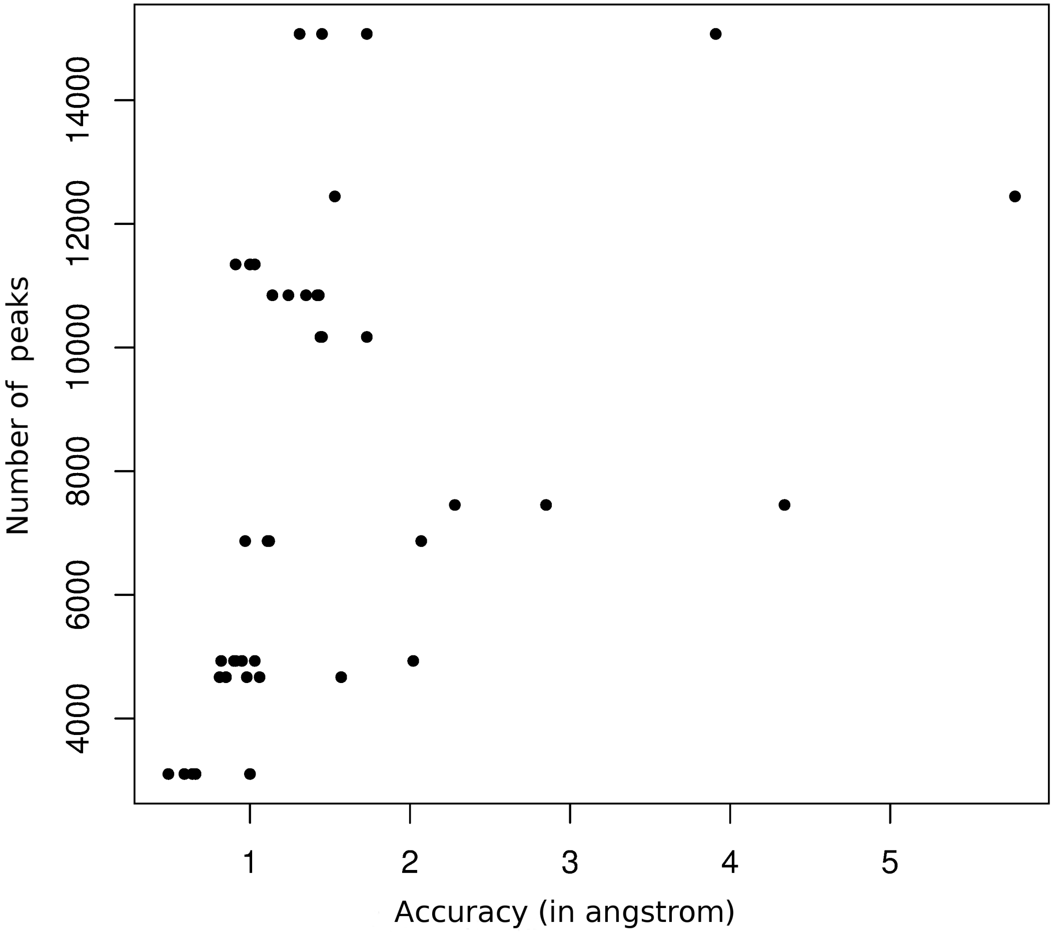

Supplementary Figure S1: Correlation between the number of un-curated ^{13}C-NOESY peaks and the structure accuracy for the un-curated methods.

Supplementary Table S3: Pearson correlation coefficients between the final structure accuracy per entry (starting from raw, un-curated or curated data) and the original number of un-curated peaks, the protein length and the number of chemical shifts.

	Raw	Un-curated	Curated
^{15}N-NOESY peaks	0.585	0.390	0.275
^{13}C-NOESY peaks	0.469	0.398	0.403
Aromatic ^{13}C-NOESY peaks	0.290	0.110	-0.168
Protein length	0.465	0.134	-0.065
CS (#)	0.494	0.164	-0.005
